# From thigh to pelvis: female genital prolapse repair with an autologous semitendinosus tendon transplant: Data of the German multicenter trial

**DOI:** 10.1007/s00192-023-05512-6

**Published:** 2023-05-02

**Authors:** Amadeus Hornemann, Tobias Weissenbacher, Benjamin Hoch, Wolfgang Franz, Neelam Lingwal, Marc Suetterlin, Bernd Holthaus

**Affiliations:** 1 Krankenhaus Sachsenhausen, Schulstraße 31, 60594 Frankfurt am Main, Germany; 2 MIC-Zentrum-München, Terminalstraße Mitte 18, 85356 München, Germany; 3 Lutrina Klinik, Brüsseler Straße 7, 67657 Kaiserslautern, Germany; 4 Department of Biostatistics and Mathematical Modelling, Goethe-University Frankfurt, Theodor-Stern Kai 7, 60590 Frankfurt am Main, Germany; 5 Medical Faculty Mannheim of the University of Heidelberg, University Medical Center Mannheim, Theodor-Kutzer-Ufer 1–3, 68167 Mannheim, Germany; 6 St. Elisabeth Krankenhaus Damme, Lindenstraße 3, 49401 Damme, Germany

**Keywords:** Semitendinosus tendon, Genital prolapse, Pelvic organ prolapse, Uterine prolapse, HoTT®, Vaginal prolapse

## Abstract

**Abstract:**

**Introduction and hypothesis:**

The use of synthetic mesh for prolapse and incontinence surgery is discussed controversially and in several countries is either no longer used or permissible. Previous approaches with autologous tissue did not show from a patient´s perspective convincing long-term results. As there have been repeatedly significant complications with synthetic mesh, a new approach is urgently needed.

During orthopedics and trauma surgeries, tendons from the thigh have been used for decades to replace cruciate ligament. The procedure of tendon removal from the thigh is fast, easy to learn and morbidity is low. In addition, a long-term durability of the transplant ought to be expected. The objective of this investigation was to show our experience with a semitendinosus tendon instead of a mesh for genital prolapse repair.

**Method:**

After the first successful attempts using such tendons in cervicosacropexy and pectopexy in patients with genital prolapse, we initiated a national multicenter study in 2020. Five German hospitals participated in order to determine the feasibility of cervicosacropexy with tendon tissue instead of mesh.

**Result:**

Up until now, we have operated and observed 113 patients for at least 6 months and have seen stable results in terms of fixation of the apical compartment. The expected low morbidity at the donor site was also confirmed through subjective assessment of the patients (Knee and Osteoarthritis Outcome Score). Improvement of quality of life was confirmed after the procedure with the Short Form Health Survey 12, Version 2.0. The results of this multicenter study showed that the desired elevation of the apical compartment with tendon tissue can be achieved with low morbidity and without a synthetic mesh.

**Conclusion:**

Women with uterine prolapse can be treated minimally invasively and with very low morbidity by using the semitendinosus tendon. The involvement of multiple (five) medical centers confirms that the technique is easy to learn and be transferred to other clinical centers.

## Introduction

Parity, vaginal births, higher age, a high body mass index (BMI) amongst other causes are risk factors for developing a genital prolapse [1]. As conservative approaches usually only alleviate the symptoms in the early stages, surgical procedures were developed. Interventions using autologous tissue usually did not lead to convincing results [2]. Different approaches using biological grafts such as fascia lata or fascia of the musculus rectus abdominis are reported as an alternative option. However, harvesting of the tissue is considered to be too invasive for implementation into a clinical routine [3]. As a result, synthetic mesh, known from hernia surgery, found its way into pelvic floor surgery by the end of the 1990s [4]. The use of synthetic mesh led to very good stability in prolapse surgery. Consequently, new types of mesh were designed, also for vaginal operations. Through a period of time new types of complications occurred, some of them very severe. The rate of postoperative pelvic pain is said to be up to 30% after placement of transvaginal tape or mesh [5, 6]. It is also described that after a synthetic mesh has been inserted, it cannot always be removed completely after a short time [7]. Implanted mesh itself appears to have a negative impact on health as well. Rheumatologists were able to show that there is a correlation between the implantation of synthetic mesh and the development of articular rheumatism [8]. These findings led to much controversy as to whether or not synthetic mesh should be further used in prolapse surgery. Warnings from the FDA in 2008 and 2011 and advice from the National Health Service (NHS) caused some manufacturers to stop producing vaginal mesh [9, 10]. The use of synthetic mesh is now banned in several countries (such as the UK, Australia, New Zealand) and reliable surgical alternatives are urgently needed [11, 12]. During urogynecological consultation patients are increasingly asking for alternative surgical methods.

Colleagues in orthopedics and trauma surgery have faced similar problems in the past [13, 14]. In addition to intolerance to the synthetic material used, material fatigue proved to be disadvantageous in cruciate ligament surgery. In contrast, surgery with autologous hamstring tendon tissue showed convincing results for the replacement of the anterior cruciate ligament [15]. Nowadays, tendon tissue is considered standard for this surgical procedure. In 2004, a very gentle method of tendon harvesting was developed. The method is easy to learn, takes only a few minutes and has excellent cosmetic results [15, 16]. Decades of experience have been collected using tendon tissue in knee surgery. In addition to the excellent durability, the good tolerability is also convincing. The morbidity caused by the loss of the tendon is so low that the tendon can even be removed from the affected leg in the event of a cruciate ligament tear. Some orthopedic surgeons even remove the gracilis tendon in addition to the semitendinosus tendon without observing significantly increased morbidity [17]. Studies with MRI showed that in the area of the removed tendon, corresponding tendon-like structures will be detected after only 2 years. The tissue seems to at least partially grow back [18, 19]. When using tendon tissue in gynecological operations, we were able to prove that only half the width of the tendon is required [20]. After removing half of the width of the semitendinosus tendon, the remaining part of the tendon can be palpated thinner, but with the same tension and identical functionality. By adapting this technique, we have indications that the morbidity in the leg can be reduced even further. Knee surgeons will be able to evaluate the amount of morbidity reduction by this modification scientifically in the very near future.

The first patient with prolapse symptoms where we used a tendon tissue instead of a synthetic mesh received cervicosacropexy [21]. The result was good, but the type of fixation on the promontory was not considered ideal. The fibrous structure of the tendon required the use of spiral staples in addition to a suture. For this reason, with the next patients a pectopexy was performed, where the tendon could be moved around the pectineal ligament on both sides [22]. Compared with sacropexy, pectopexy seems to be effective too, but has not yet been established internationally. Critics acknowledged the use of the tendon tissue as innovative but looked at the pectopexy itself critically and as a result the international perception of the procedure was limited.

Based on this experience, we wanted to continue evaluating the feasibility of the technique systematically throughout several hospitals in Germany. The medical study was based on the hypothesis that the surgical technique is easy to learn and should easily be adopted by other laparoscopic gynecological surgeons. In parallel, we further optimized and standardized the technique of sacropexy with tendon tissue and improved the fixation on the promontory. As of now, we have used tendon tissue as a substitute for a tension-free vaginal tape (TVT) in several patients with stress urinary incontinence and have achieved satisfactory results [23].

In the following we present the preliminary results of our national multicenter trial in 113 patients after sacropexy using a tendon tissue transplant including a 6-month follow-up.

## Materials and methods

Patients who were referred for surgical treatment of a symptomatic mild (POP-Q stage 2) to severe (POP-Q stage 3–4) uterine, cervical or vaginal prolapse were offered the option of using tendon tissue instead of a synthetic mesh for sacropexy. If they showed interest in this alternative methodology, they were included in the study. Criteria for the investigation was a symptomatic prolapse of the apical compartment in patients older than 18 years. Conservative treatment was evaluated to be insufficient or it was not accepted by the patient. Patients with anamnestic bilateral cruciate ligament surgery with hamstring transplant and patients with a contraindication for laparoscopy were excluded. After a detailed medical briefing and a written declaration of consent, the minimally invasive surgery was performed, with a sacropexy being the central surgical step. For the laparoscopic operation straight stick instruments were used. Depending on the findings during surgery, in some cases further surgical interventions needed to be performed, such as supracervical hysterectomy with salpingo-oophorectomy, laparoscopic anterior colporrhaphy, or adhesiolysis. The thigh (left or right) from which to harvest the tendon tissue was determined together with the patient, after an in-depth medical briefing. For the purpose of this study, there was no favorite side.

The main result of this study was to prove the feasibility of the procedure in an adequate time, with low morbidity and with a comparable outcome with the use of synthetic mesh in these types of operations. The operators were laparoscopically trained gynecologists with experience in laparoscopic mesh surgery. As harvesting of the tendon tissue is a very easy procedure, no specific training was necessary.

**Fig. 1:**
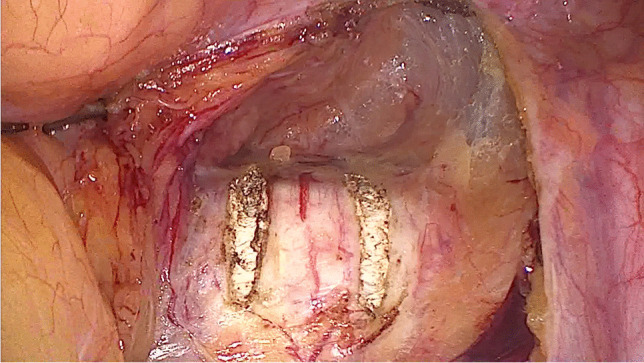
Longitudinal ligament with two parallel incisions at the level L5/S1

**Fig. 2:**
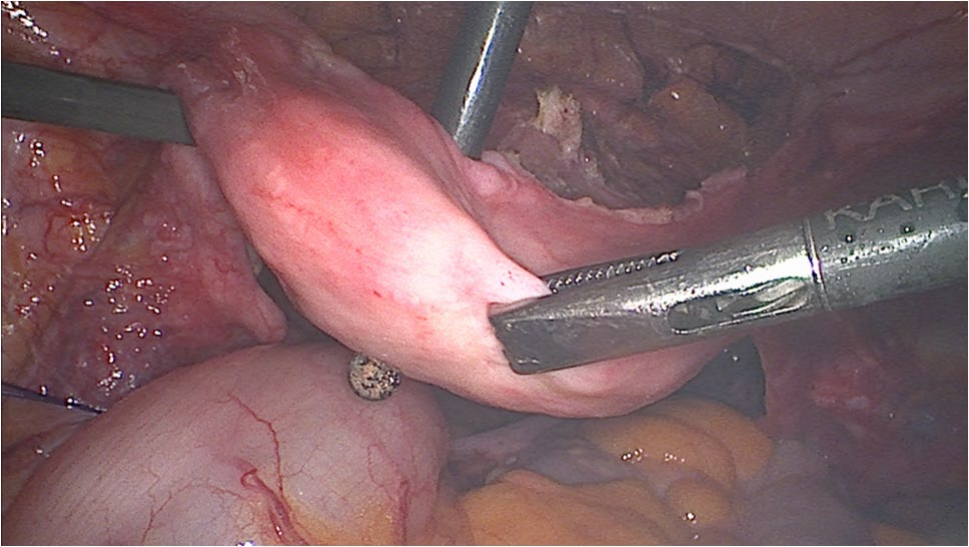
Perforating the cervix with a monopolar spatula (hysterosacropexy)

**Fig. 3:**
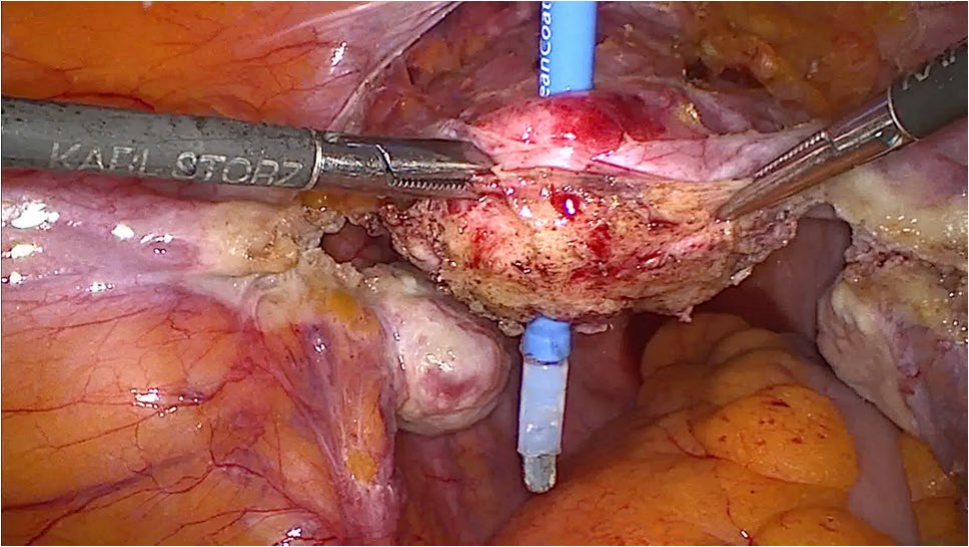
Perforating the cervix with a monopolar spatula (cervicosacropexy)

After disinfection of the abdominal wall and the selected leg, laparoscopy was performed first. Among other things, the longitudinal ligament was prepared at the level of the promontory, with two parallel incisions of approximately 25 mm and a distance of approximately 10 mm (Fig. 1). As large blood vessels could potentially be found in this area, extensive exposure of the ligament is mandatory. Then, after possibly necessary preparatory surgical steps, the structure to be elevated (vagina, cervix, or uterus) was exposed for fixation and, in the case of hystero- or cervicopexy, was perforated in two parallel places with a monopolar spatula (Medtronic, Minneapolis, MN, USA) (Figs. 2, 3).

**Fig. 4:**
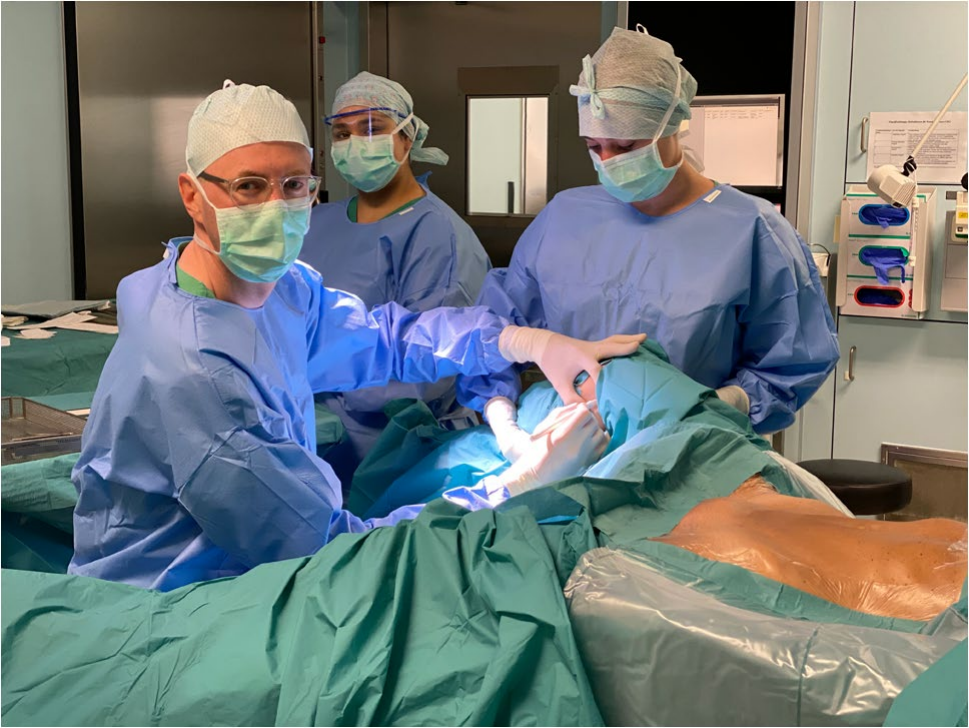
Marking the back of the right knee before the skin incision

**Fig. 5:**
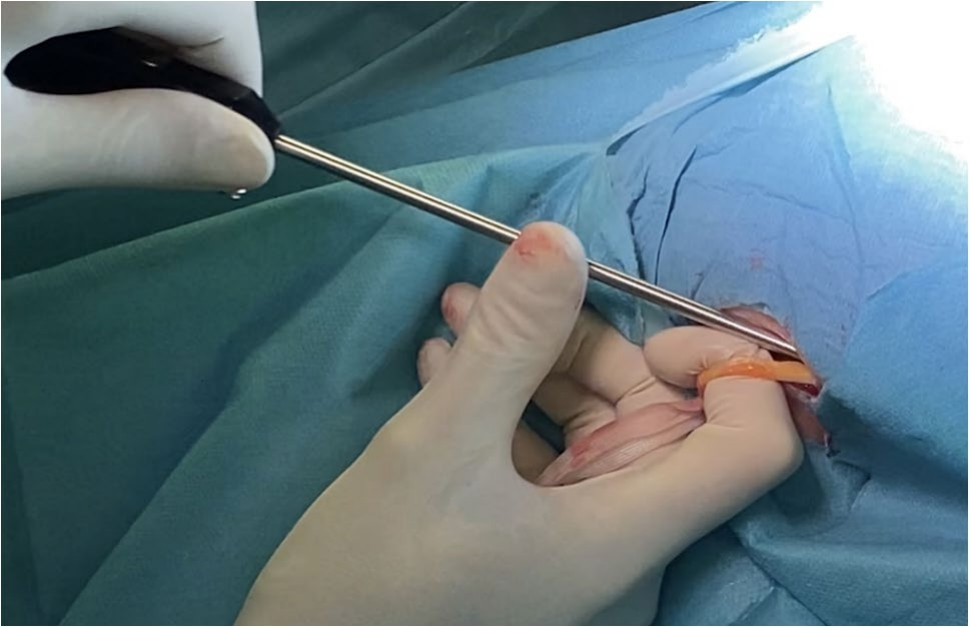
Removing the right semitendinosus tendon with a tendon stripper

**Fig. 6:**
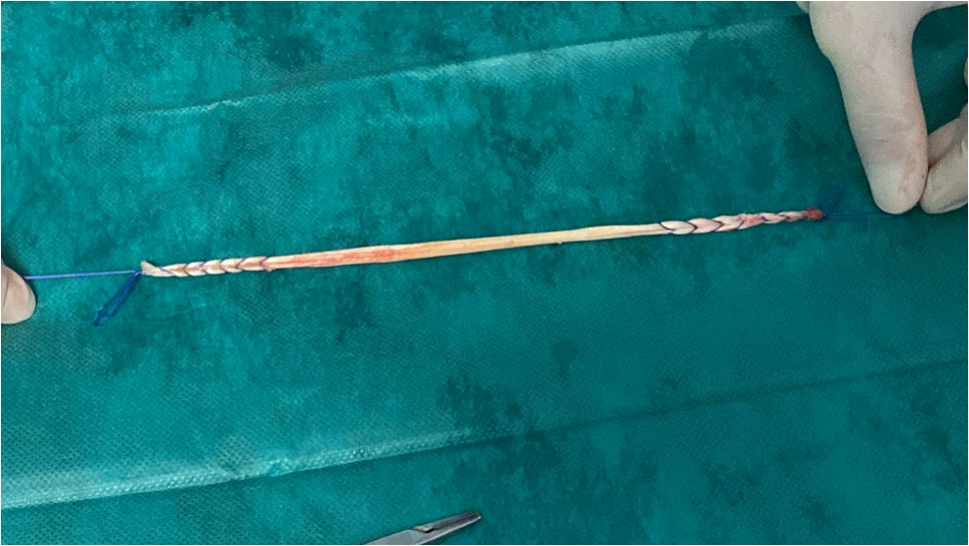
Both ends of the tendon are prepared extracorporeally with absorbable sutures in case of hystero- and cervicosacropexy (HoTT®-Sling, Set 2+1; SMI, 4780 St. Vith, Belgium) or non-absorbable sutures in case of vaginosacropexy (HoTT®-Sling, Set 3; SMI, 4780 St. Vith, Belgium)

**Fig. 7:**
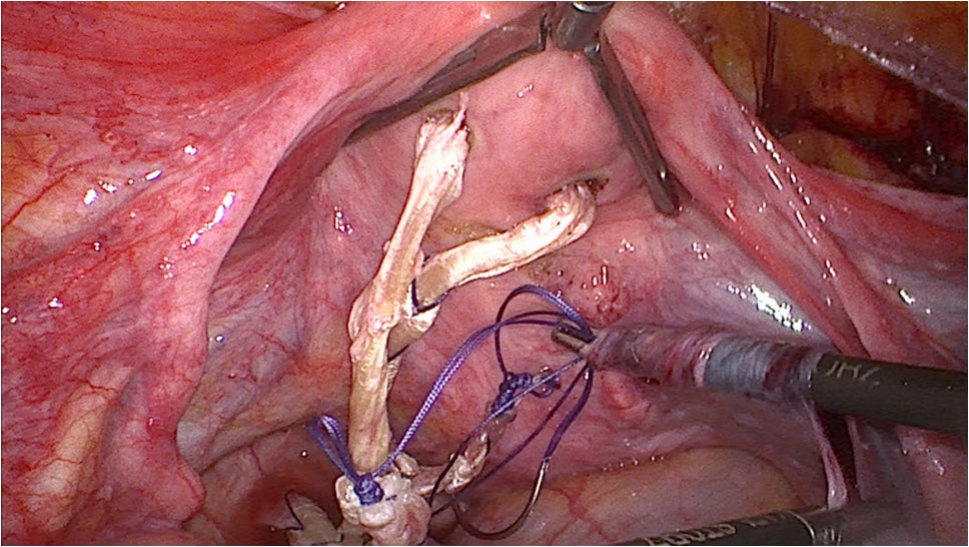
The tendon is pulled through the perforations of the uterus (hysterosacropexy)

**Fig. 8:**
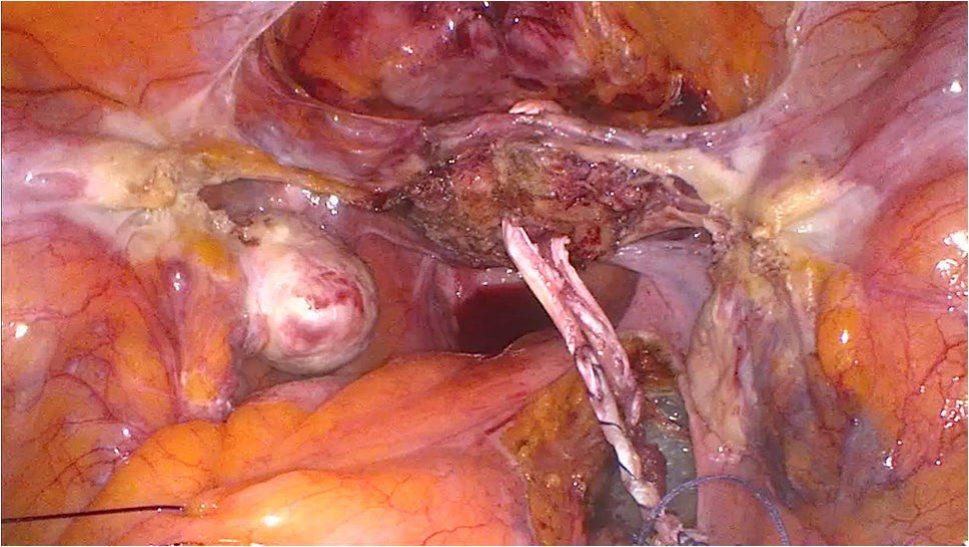
The tendon is pulled through the perforations of the cervix (cervicosacropexy)

**Fig. 9:**
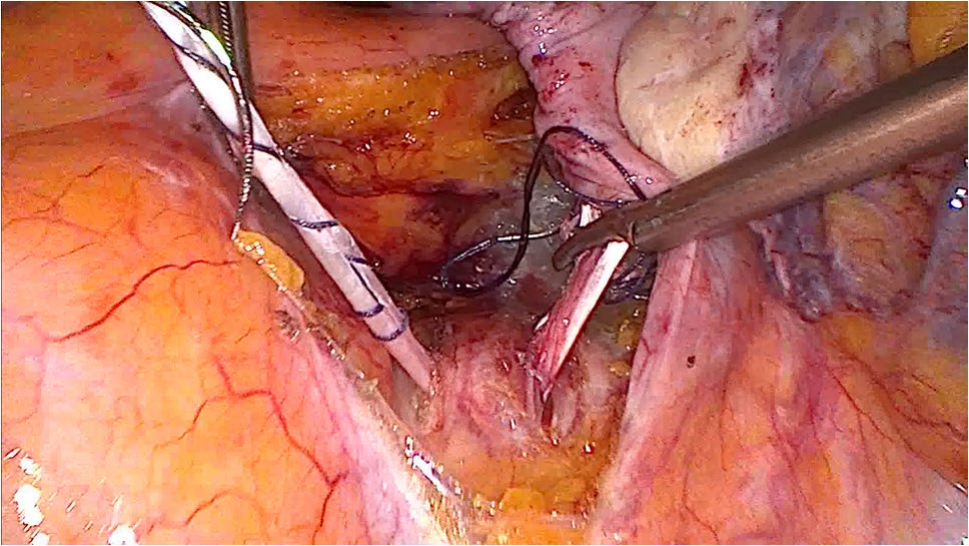
The tendon is pulled through the longitudinal ligament

**Fig. 10:**
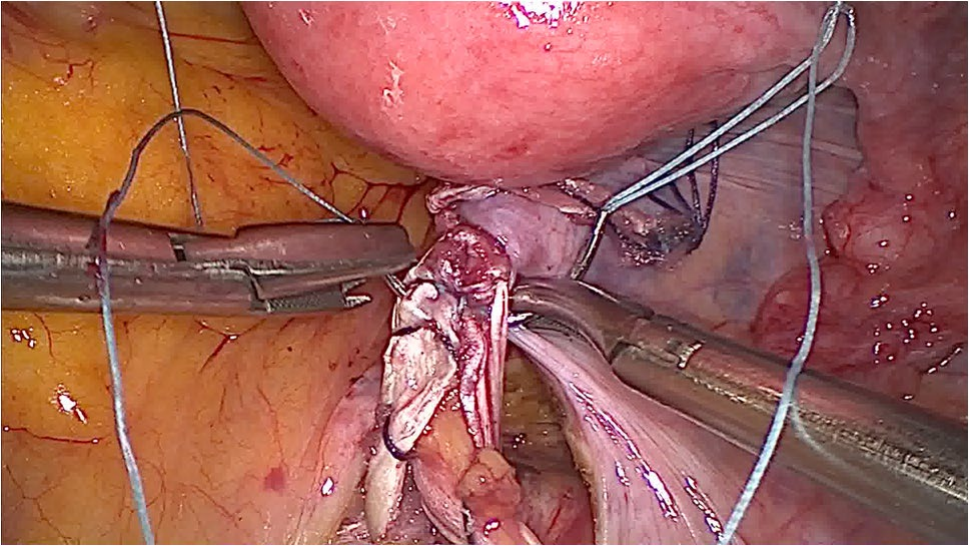
Suturing the ends of the tendon together (side to side) (HoTT®-Sling, SMI, 4780 St. Vith, Belgium)

**Fig. 11:**
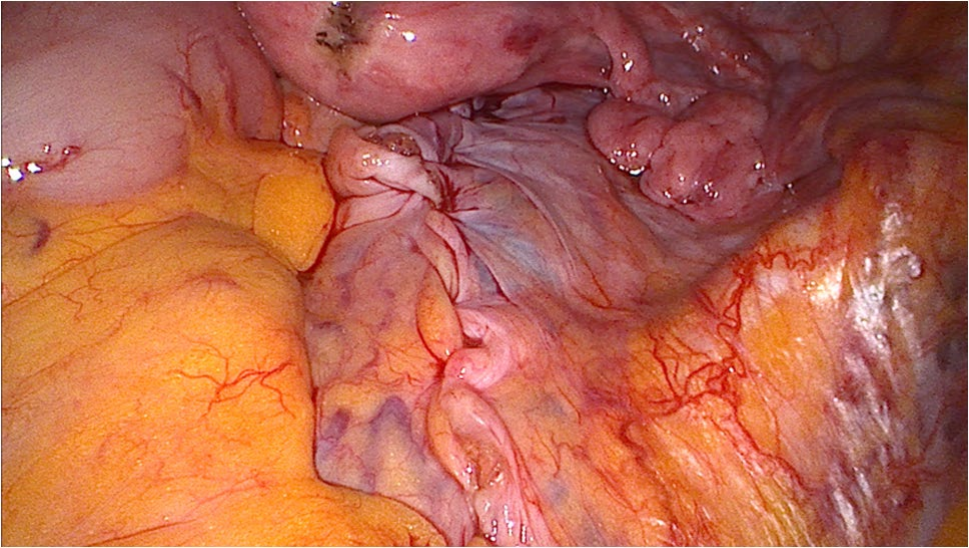
Situation after closing the peritoneum with an absorbable suture

After the sacropexy was proved to be feasible, half of the width of the tendon was removed from the thigh (Figs. 4, 5). This was done with an approximately 25- to 30-mm horizontal skin incision in the back of the knee. After the tendon of the semitendinosus muscle was clearly identified, the tendon was incised lengthwise down the middle. This allows half of the tendon only to be detached from the semitendinosus muscle by using a blunt tendon stripper [20] (Arthrex, Naples, FL, USA). The tendon tissue was then handed over to the surgical nurse and the skin closed with two single stitches. Drainage was not used in any of the patients and only one required hemostasis. Both ends of the tendon were prepared extracorporeally with absorbable sutures (Fig. 6) (HoTT®-Sling; SMI, St. Vith, Belgium). The tendon was then introduced into the abdomen via the trocar. In the case of hystero- or cervicosacropexy, the tendon was pulled from dorsal to ventral and back through the perforations (Figs. 7, 8). Then it was pulled through the incised longitudinal ligament (Fig. 9) and both ends were fixed together with a non-absorbable suture (Fig. 10) (HoTT®-Sling; SMI). In the case of a colpopexy, the tendon was sutured with a non-absorbable suture dorsally to the vagina, pulled through the incisions of the longitudinal ligament, and then sutured ventrally to the vagina. The final step was the closure of the peritoneum (Fig. 11).

All patients went through a standardized gynecological follow-up examination by their surgeon, first after 6 weeks and finally 6 months after the surgery. During these examinations the POP-Q Score was measured and documented for the apical compartment. Before the operation, after 6 weeks, and after 6 months, the patients received standardized questionnaires (Knee and Osteoarthritis Outcome Score, KOOS [24] and the Short Form Health Survey 12, Version 2.0, SF-12-v2 [25]). These questionnaires are validated for health in general as well as for the functionality of the knee.

The Ethics Committee II of the University of Heidelberg approved the study (2018-602N-MA). This was also confirmed by the ethics committees of the Hessian State Medical Association, the Lower Saxony State Medical Association, and the Bavarian State Medical Association.

For the evaluation of data, the patients received standardized questionnaires (KOOS [24] and SF-12-v2 [25]). The descriptive analysis of quantitative variables included (a) mean, (b) median, and (c) interquartile intervals. The Friedman test with post hoc Wilcoxon signed-rank tests was used for the data evaluation on the respective scales for chronological comparison (at baseline and at 6 weeks and 6 months). Data obtained in the study were analyzed using statistic software BiAS (Version 10.04; EPSiLON, 1989–2013), and statistical level of significance was set at p < 0.05 Table 1.

**Table 1:** Knee and Osteoarthritis Outcome Score questionnaire (0 = worst, 100 = best)

	Before the operation, mean (SD)	After 6 weeks, mean (SD)	After 6 months, mean (SD)	*p* value
Symptoms	94.3 (8.7)	92.2 (11.1)	93.8 (11)	0.856
Knee pain	96.6 (8.4)	90.8 (13.3)	96.5 (8.4)	<0.0001
Activity of daily life	96.4 (8.7)	92.1 (13)	97 (6.3)	<0.0001
Sport/recreation	91.9 (16.9)	85.1 (20.2)	91.8 (16)	<0.0001
Quality of life	92.7 (16.1)	87.4 (18.5)	93.7 (11.8)	<0.0001

## Results

A total of 91 patients received cervicosacropexy, 10 patients hysterosacropexy, and 12 patients vaginosacropexy. All operations were performed successfully. The average age was 58 years (31–81 years). The average BMI was 24 kg/m^2^ (18–35 kg/m^2^). The average incision-to-suture time for the entire operation was 123 min (88–233 min). The mean incision-to-suture time for tendon harvest was 8 min (range 3–23 min). Blood loss was very low (mean 82 ml, range 0–200 ml).

**Fig. 12:**
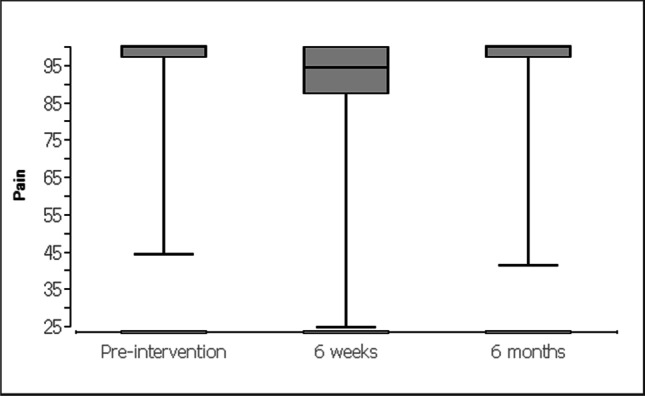
Knee and Osteoarthritis Outcome Score concerning knee pain

**Fig. 13:**
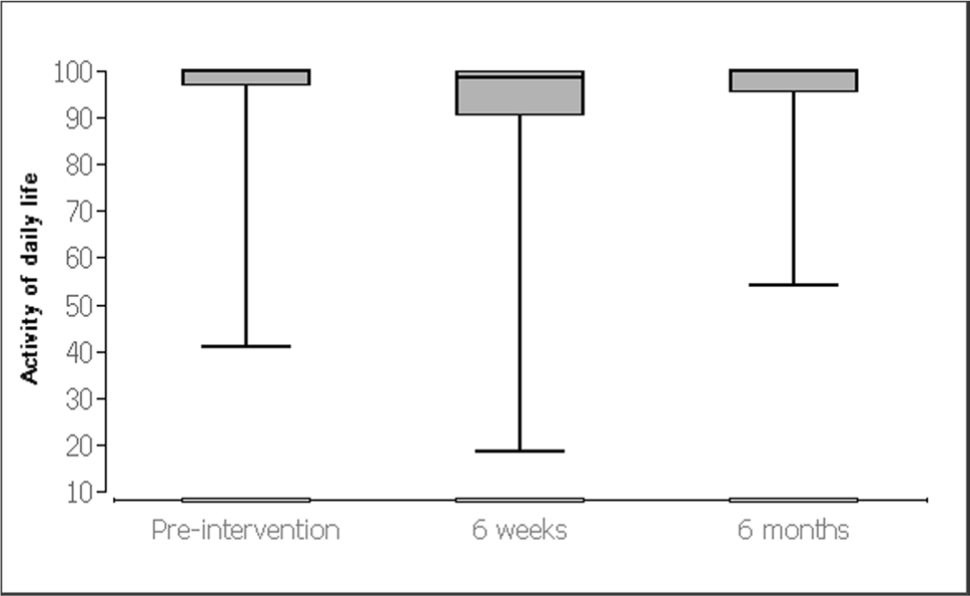
Knee and Osteoarthritis Outcome Score concerning activity of daily life

**Fig. 14:**
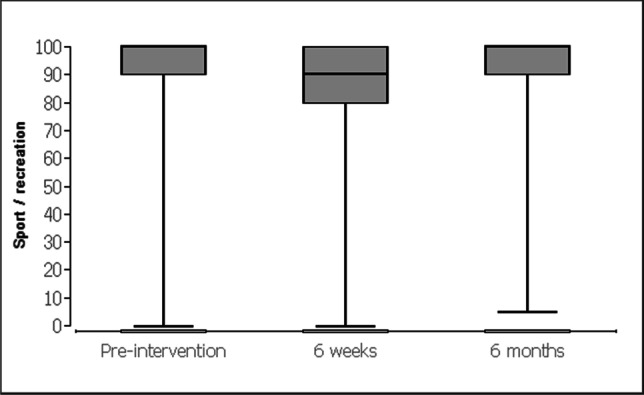
Knee and Osteoarthritis Outcome Score concerning sports

**Fig. 15:**
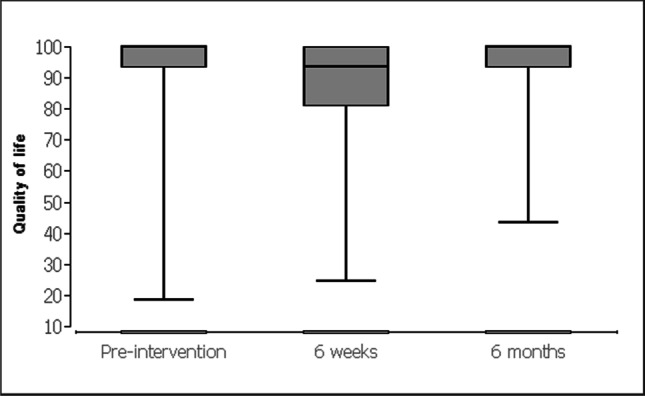
Knee and Osteoarthritis Outcome Score concerning quality of life

Prior to the surgery the apical compartment (C-compartment) was described with a POP-Q stage between 2 and 4 (mean 2.55). Stage 0 was achieved after the operation in all cases by fixation. The controls after 6 weeks and after 6 months showed no change compared with the immediate postoperative result (stage 0). After 6 weeks, the KOOS questionnaire (Table 2) showed a higher pain level (Fig. 12) and lower scores concerning activity of daily life (Fig. 13), sport (Fig. 14), and quality of life (Fig. 15). After 6 months, the values for activity of daily life and quality of life showed some improvement compared with the initial values (Table 1). Physical activity was unaffected by the loss of the tendon and showed the same values after 6 months as before the operation (Table 1). To assess quality of life before and after, patients completed the SF-12-v2. Several of the 12 questions showed significant differences in quality of life. All parameters showed improvement after 6 months (Table 2).

**Table 2:** Short Form Health Survey 12, Version 2.0

	Before the operation, mean (SD)	After 6 weeks, mean (SD)	After 6 months, mean (SD)	*p* value
In general, would you say your health is: good (1) to bad (5)	2.6 (0.8)	2.5 (0.8)	2.3 (0.8)	0.353
The following questions are about activities you might do during a typical day. Does your health now limit you in these activities? If so, how much?				
Moderate activities: severe influence (1) to no influence (3)	2.5 (0.6)	2.4 (0.6)	2.7 (0.5)	<0.001
Climbing several flights of stairs: severe influence (1) to no influence (3)	2.5 (0.6)	2.5 (0.6)	2.7 (0.5)	0.018
During the past 4 weeks, how much of the time have you had any of the following problems with your work or other regular daily activities as a result of your physical health?				
Accomplished less than you would like: always (1) to never (5)	3.7 (1.1)	3.5 (1.1)	4.2 (0.9)	<0.01
Limited in the kind of work or activities: always (1) to never (5)	3.8 (1.1)	3.7 (1.1)	4.3 (0.8)	<0.01
During the past 4 weeks, how much of the time have you had any of the following problems with your work or other regular daily activities as a result of any emotional problems (such as feeling depressed or anxious)?				
Accomplish less than you would like: always (1) to never (5)	3.9 (1.2)	4 (1)	4.3 (0.8)	0.068
Didn’t do work or other activities as carefully as usual: always (1) to never (5)	4 (1.1)	4.1 (1)	4.4 (0.9)	0.134
How much did pain interfere with normal work: not (1) to very much (5)	1.9 (1.1)	1.9 (1.1)	1.2 (0.5)	<0.0001
These questions are about how you feel and how things have been with you during the past 4 weeks. For each question, please give the one answer that comes closest to the way you have been feeling. How much of the time during the past 4 weeks				
Felt calm and peaceful: always (1) to never (5)	2.3 (0.9)	2.1 (0.8)	1.9 (0.7)	<0.001
Have a lot of energy: always (1) to never (5)	2.5 (1)	2.4 (1)	2.1 (0.7)	0.157
Felt downhearted and depressed: always (1) to never (5)	3.8 (1)	4 (1)	4 (1)	0.16
How much time health interferes with social activities: always (1) to never (5)	4.2 (1)	4.2 (1)	4.5 (0.7)	<0.01

Two bladder injuries occurred intraoperatively and were repaired immediately. No late complications of the interventions were detected. After 6 months, one patient showed a clearly isolated cystocele, whereas the cervix was still well fixed. This cystocele was corrected in a vaginal procedure.

In most patients, superficial ecchymosis of the skin was observed in the area of ​​tendon harvesting (thigh and lower leg). This regressed without intervention and was no longer detectable after 6 weeks. There were no functional limitations of the leg or severe infections.

## Discussion

The purpose of this study was to evaluate the feasibility and safety of laparoscopic sacropexy using tendon tissue of the thigh in a medical multicenter setting. This goal was confirmed successfully. A semitendinosus transplant was used and was performed well in all patients. The mean operating time was 123 min, with a large variability owing to the number of potentially additionally needed operative procedures. Even though these are already satisfactory surgery times, it was observed throughout the 113 medical cases that the mean surgical time for tendon harvesting and sacropexy will be further reduced with increased practice. The mean duration of tendon harvesting (cut-to-suture time) in our study was 8 min. With increasing practice, we have been able to shorten this to less than 10 min in every patient. The implantation of tendon tissue was possible in all 113 patients. Based on our study, we also recommend removing the tendon in the future as the first step and not during laparoscopy.

We used the KOOS questionnaire to evaluate the morbidity in the leg before and after the operation. It serves as an evaluated and standardized instrument to describe the functionality in the knee joint. The questionnaire is less precisely applicable for the leg situation after harvesting of tendon tissue. Overall, it could be shown that almost no effects on the knee joint were observed. Conversations with all patients did prove that the procedure did not cause any relevant leg problems. The morbidity at the donor site was low and there were no functional limitations. It was observed that patients still felt symptoms of the operation after 6 weeks (Table 1). After a total of 6 months, however, all patients felt significantly better than before the operation. The functionality of the operated leg was equal to the situation before the operation (Tables 1, 2). After 6 months, some of the patients were not even able to remember from which leg the tendon had been removed. Our results for the morbidity in the leg also confirm the results of orthopedic studies on the functionality of the leg after tendon transplantation for anterior cruciate ligament replacement [16].

An isolated cystocele was seen in one patient after 6 months. This could be corrected with vaginal anterior colporrhaphy. A cystocele can occur after sacropexy because the connective tissue in these patients is weakened overall in the pelvic floor area. The anterior compartment is particularly exposed to intra-abdominal pressure after sacropexy of the apical compartment. Corresponding findings have also been described in the past after cervicosacropexy using the conventional, plastic-based technique [26].

Cruciate ligament lesions are usually seen in physically active and significantly younger patients. Our results showed that the low morbidity due to removal of the tendon of the semitendinosus muscle can also be seen in older people.

The use of synthetic meshes in prolapse surgery is controversial and more and more patients are asking for alternative options [2]. The use of tendon tissue combines two established surgical methods. The implementation also showed good results in our medical multicenter setting. As a result, we recommend further evaluation of the method in studies and see the possibility of offering this quality-controlled alternative to the use of a synthetic mesh in the near future.

One of the limitations of the study was that, even with a follow-up period of 6 months, long-term results are still pending. Knee surgeons expect the tendon as a replacement for the cruciate ligament in the knee joint to last for a lifetime, but there has been no proof for the abdominal area until now. Furthermore, the operations were performed by particularly well-trained doctors with a high level of expertise. Studies with even more centers must prove that the technique can also be performed by less experienced surgeons. As the use of tendon tissue is a new technique, a selection bias by choosing the right patients may not be entirely excluded. Further investigations with even more patients will show whether our results are transferrable.

## Conclusion

The use of the semitendinosus tendon for sacropexy is a safe, fast, and easy-to-learn procedure, is of a minimally invasive nature, and provides convincing short- and medium-term results, according to the preliminary analysis of our study.
